# The Influence of Alumina Airborne-Particle Abrasion with Various Sizes of Alumina Particles on the Phase Transformation and Fracture Resistance of Zirconia-Based Dental Ceramics

**DOI:** 10.3390/ma16155419

**Published:** 2023-08-02

**Authors:** Paulina Łagodzińska, Beata Dejak, Michał Krasowski, Bartłomiej Konieczny

**Affiliations:** 1 Department of Prosthodontics, Medical University of Lodz, 92-209 Łódź, Poland; 2 University Laboratory of Material Research, Medical University of Lodz, 92-209 Łódź, Poland

**Keywords:** zirconia, 3Y-TZP, airborne-particle abrasion, alumina, phase transformation, fracture resistance

## Abstract

The surface of zirconia-based dental ceramic restorations require preparation prior to adhesive cementation. The purpose of this study was to assess the influence of airborne-particle abrasion with different sizes of alumina particles (50 μm, 110 μm, or 250 μm) on the mechanical strength of zirconia-based ceramics’ frameworks and on the extent of phase transformations. A fracture resistance test was performed. The central surface of the frameworks was subjected to a load [N]. The identification and quantitative determination of the crystalline phase present in the zirconia specimens was assessed using X-ray diffraction. The Kruskal–Wallis one-way analysis of variance was used to establish significance (α = 0.05). The fracture resistance of zirconia-based frameworks significantly increases with an increase in the size of alumina particles used for air abrasion: 715.5 N for 250 μm alumina particles, 661.1 N for 110 μm, 608.7 N for 50 μm and the lowest for the untreated specimens (364.2 N). The X-ray diffraction analysis showed an increase in the monoclinic phase content after air abrasion: 50 μm alumina particles—26%, 110 μm—40%, 250 μm—56%, and no treatment—none. Air abrasion of the zirconia-based dental ceramics’ surface with alumina particles increases the fracture resistance of zirconia copings and the monoclinic phase volume. This increase is strongly related to the alumina particle size.

## 1. Introduction

Zirconia-based dental ceramics possess superior mechanical parameters to other types of dental ceramics. They are widely used in dentistry, particularly in prosthodontics, as the material for full-ceramic dental crowns and bridge frameworks, monolithic restorations, and implant suprastructures. However, the adhesive bonding of 3Y-TZP restorations (3 mol % yttria stabilized tetragonal zirconia polycrystals) to resin cements is impaired by the structure and inertness of zirconia [1,2,3]. A number of surface treatments have been recommended in the literature to increase the bond strength of zirconia-based ceramics to resin cements. The method of choice is airborne-particle abrasion with alumina particles and the application of primers incorporating organophosphate monomers [2,3,4,5,6,7,8,9,10]. Laboratory tests indicate that this approach significantly improves the bonding capacity [4,10,11,12,13,14]. In addition, airborne alumina abrasion guarantees micro-mechanical retention, activates the surface, increases wettability, and ensures proper cleaning of organic and inorganic contamination from the 3Y-TZP surface [15].

Alumina airborne-particle abrasion, besides its obvious advantages, may also cause defects in the topmost surface layer of 3Y-TZP, such as microscopic cracks, grooves, the plastic deformation of crystals, and the embedding of alumina particles [16,17,18,19,20,21]. It also affects the phase content and induces a tetragonal-to-monoclinic phase transformation [16,17,18,19].

Pure zirconia is a polymorphic crystalline material that presents three allotropic forms depending on the temperature of the environment: monoclinic (*m*), tetragonal (*t*), and cubic (*c*) phases. At room temperature, zirconia is composed of the monoclinic phase. It transforms into tetragonal upon heating above 1170 °C; at 2370 °C, it transforms into the cubic phase, and it melts above 2716 °C [22]. Cooling results in a tetragonal-to-monoclinic phase transformation (*t* → *m*). This transition results in a 4–5% expansion of crystals’ volume, causing the formation of surface compressive stresses [22,23,24,25]. The addition of dopants such as MgO, CaO, Y_2_O_3_, and CeO_2_ results in the formation of thermodynamically stable and more reliable tetragonal zirconia, which is not prone to spontaneous phase transformations. Therefore, zirconia-based dental ceramics are usually stabilized by 3% mol yttria (3Y-TZP). Two phenomena are associated with the *t* → *m* transformation: PTT, phase transformation toughening, and LTD, low temperature degradation [22,26].

PTT results in an immediate increase of mechanical strength of zirconia-based ceramics. It is usually triggered by mechanical stimuli such as grinding or air abrasion, which induce the t → m transformation and volumetric expansion. If any crack or fracture propagates from the surface, its passage is blocked by this increase in crystal volume [25,27].

Phase transformations are also connected with the LTD phenomenon [27]. In this case, the t → m transformation happens slowly and is usually triggered by constant mechanical stress stimulation and moisture [28]. It can also be affected by the zirconia grain size, superficial degradation of the zirconia surface, temperature (20–250 °C), sintering process parameters, as well as the content and distribution of dopants [26,28]. In vitro laboratory studies demonstrate that LTD may be detrimental to mechanical and aesthetic parameters of 3Y-TZP. Also, it leads to zirconia grain pull-out, as well as an increase in the roughness and occurrence of internal stresses or surface erosion [22,26,29]. PTT is strongly connected with the LTD phenomenon, as it alters the phase content and the integrity of the material structure; hence, such restoration may be susceptible to LTD, and PTT affects its intensity [27]. The tetragonal-to-monoclinic transformation that had occurred in the material is irreversible at mouth temperature [27]. Only regeneration firing, such as in a porcelain furnace, may reverse this transformation [30]. Recent research, however, indicates that airborne-particle abrasion with alumina particles may not affect the stability of zirconia ceramics and may possibly even enhance their resistance to LTD compared to an intact 3Y-TZP surface [31,32].

As mentioned before, prosthetic restorations made of zirconia-based dental ceramics require special treatment before the application of resin cements. The method of choice is alumina air abrasion of the surface of the restoration followed by coating with primers. Air abrasion with alumina particles is an effective and widely available method of improving the bonding capacity of zirconia-based restorations. However, it may induce a t–m transformation (strongly connected with the LTD and PTT phenomena) and influence the mechanical parameters of zirconia. The conditions of air abrasion such as the size of alumina particles may influence the extent of the zirconia surface alterations.

The purpose of this study was to assess the influence of airborne-particle abrasion with various sizes of alumina particles (50 μm, 110 μm, and 250 μm) on the fracture resistance of zirconia-based frameworks and on the extent of the t–m phase transformation. The null hypothesis was that the use of various sizes of alumina particles for the mechanical abrasion of 3Y-TZP does not affect its mechanical properties such as the fracture resistance of frameworks or a tetragonal-to-monoclinic transformation.

## 2. Materials and Methods

The blanks of commercial pre-sintered 3Y-TZP ceramics were used in this study (Ceramill Zi; Amann Girrbach AG, Koblach, Austria) in accordance with the norm DIN EN ISO 6872:2009 and ISO 13356:1997. They were available in the form of semi-circular blocks of compressed 3Y-TZP powder intended for CAD/CAM technology (computer aided design—computer aided manufacturing). Two tests were conducted: the fracture resistance of zirconia frameworks and the X-ray diffraction assessment of phase transformations (XRD).

### 2.1. The Fracture Resistance of Zirconia Frameworks

The specimens, in the form of monolithic crowns, were fabricated in the CAD/CAM technology. The cast model was used to prepare the abutment tooth. The second molar was prepared under the control of a paralelometer (Kavo EWL 990; Kavo Polska Sp. z.o.o., Warsaw, Poland) following guidelines: 6 degrees taper, no sharp edges, and 0.5 mm—width radial shoulder margin. The occlusal surface was prepared with the concavity matching the dimensions and shape of the spherical intender of the Universal Testing Machine.

**Figure 1:**
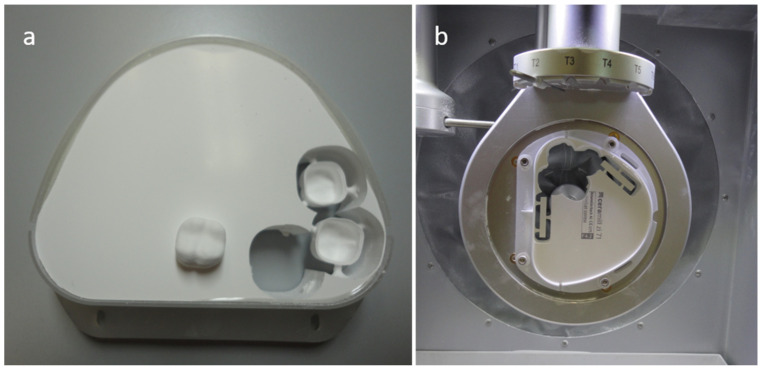
The zirconia-based dental ceramics specimens fabricated in the CAD/CAM technology. The figure shows milled samples before the sintering step: (**a**) specimens used to assess the fracture resistance of zirconia-based frameworks; (**b**) cuboid-shaped specimens used in X-ray diffraction test to assess the t–m transformation.

The cast model with the prepared abutment tooth was scanned (Ceramill Map 300; Amann Girrbach AG, Koblach, Austria). Then, the shape of the framework with uniformly thick walls (of 0.5 mm) was designed in the software (Ceramill Mind 3.11; Amann Girrbach AG, Koblach, Austria), converted into milling strips, loaded into the milling machine, and was finally dry-processed (Ceramill Motion; Amann Girrbach AG, Koblach, Austria). Sixty-eight identical specimens were milled (Ceramill Mind, Ceramill Motion; Amann Girrbach AG, Koblach, Austria). The specimens before the sintering step are shown in Figure 1a. All specimens were sintered for 10 h (Ceramill Therm; Amann Girrbach AG, Koblach, Austria). The temperature growth was 8 °C/min, starting from 200 °C to 1450 °C, with two hours of a constant temperature and then a cooling time. The material shrinkage was approximately 21%.

Specimens were randomly divided into four groups depending on the size of alumina particles used for air abrasion: 50 μm, 110 μm, and 250 μm (Alustral; Omnident Dental HandelsgesellschaftmbH, Rodgau, Germany). One group was left untreated (the control group). The inner surface of the frameworks was air-abraded at the pressure of 3 bar (Basic Classicl Renfert GmbH, Hilzingen, Germany). Prior to air abrasion, the inner surface of each framework was colored with a permanent black marker, and the exterior of the crowns was protected with Teflon tape. The air abrasion was performed in a systematic manner, recommended by Kern, until complete color removal. Such method ensures the homogenous air abrasion of the inner surface, no omission of any surface, and the protection of the exterior surface [33]. The surface of each specimen was then cleaned with an oil-free air stream [34].

The influence of the size of alumina particles used for air abrasion on thefracture resistance of zirconia-based frameworks was examined. The fracture load in each group was measured in Universal Testing Machine (Zwick Z2.5 zwickLine; Zwick GmbH & Co. KG, Ulm, Germany). Specimens were mounted on a steel basis covered with a thin silicone mat. The central occlusal surface of each specimen was subjected to the load with the spherical indenter (diameter 3.5 mm) at speed 1 mm/min. The maximum load was registered [N].

### 2.2. X-ray Diffraction

To assess the presence of a monoclinic phase in the zirconia-based specimens after alumina air abrasion, the X-ray diffraction test (XRD) was conducted. The examined surface of specimens needed to be flat. Cuboid-shaped specimens with the dimensions 5 × 5 × 20 mm were designed in the computer software (Ceramill Mind; Amann Girrbach AG, Koblach, Austria), processed to the milling device, and dry-machined (Ceramill Motion; Amann Girrbach AG, Koblach, Austria). The specimens before the sintering step are shown in Figure 1b. All specimens were sintered for 10 h (Ceramill Therm; Amann Girrbach AG, Koblach, Austria). The temperature growth was 8 °C/min, starting from 200 °C to 1450 °C, with two hours of a constant temperature and then a cooling time. The material shrinkage was approximately 21%. Eventually, specimens were 4 × 4 × 15.8 mm in dimension.

Specimens were randomly divided into four groups depending on the size of alumina particles used for air abrasion, respectively, 50 μm, 110 μm, and 250 μm (Alustral; Omnident Dental HandelsgesellschaftmbH, Rodgau, Germany). Specimens in one group were left untreated (the control group). Other specimens were air-abraded at a 45° angle, at a 13 mm distance, at a pressure of 3 bar, for 10 s (Basic Classicl Renfert GmbH, Hilzingen, Germany).

The identification and quantitative determination of crystalline phases present in zirconia specimens was conducted using the X-ray diffraction method (PANalytical Empyrean; PANalytical B.V., EA Almelo, The Netherlands) [35]. The qualitative analysis was performed with High Score Plus Programme and the ICCD PDF 4+ database. The percentage of the monoclinic phase volume in each specimen was calculated. The quantitative analysis was performed with the Chung equation method, in accordance with RIR data.

- Scans were performed using a Bragg–Brentano design in θ-θ mode with the parameters below;
- Incident beam: Co Kα radiation at 40 kV and 45 mA, a programmable divergence slit of ½ degree, a Soller slit of 0.04 rad, and a mask measuring 5 mm;
- Diffracted beam: semiconductor detector X’Celerator, a Soller slit of 0.04 rad, and a Kβ filter;
- A goniometer of five degrees of freedom: X-Y-Z-Phi-Chi;
- For the small-angle X-ray diffractograms, a 0.1 degrees/step and counting time of 100 s per step were used.

### 2.3. Statistical Analysis

The statistical analysis was performed with the Kruskal–Wallis one-way analysis-of-variance-by-ranks test, with a level of significance set at 0.05.

## 3. Results

**Figure 2:**
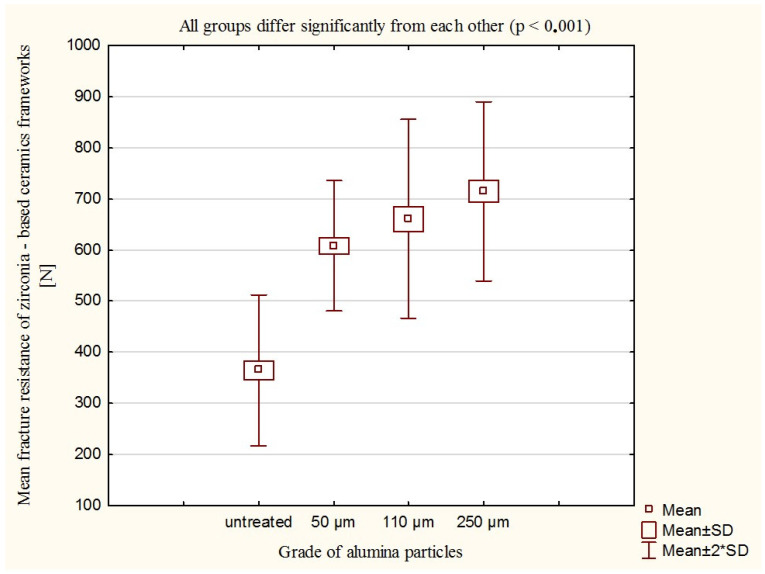
Fracture resistance of zirconia-based dental ceramics frameworks—mean values in N, standard deviations of tested groups: specimens with an untreated surface and specimens that were air-abraded with alumina particles of different sizes: 50 µm, 110 µm, and 250 µm. According to the Kruskal–Wallis one-way analysis of variance by rank test, all groups differ significantly from each other (*p* < 0.001).

The mean fracture resistance of zirconia-based frameworks values in N, and the standard deviations of all tested groups are shown in Figure 2. The fracture resistance increased with the increase of the alumina particle size used for airborne-particle abrasion, reaching the highest values for 250 µm alumina particles (715.5 N), followed by 110 µm alumina particles (661.1 N), and 50 µm (608.7 N). For the untreated specimens, the fracture resistance was 364.2 N. Air-abrading of the inner surface of the zirconia frameworks resulted in about twice higher values of fracture resistance. The Kruskal–Wallis one-way analysis-of-variance-by-rank test revealed that the use of air abrasion significantly affected the fracture resistance of zirconia frameworks (*p* < 0.001). Also, the size of alumina particles significantly affected the fracture resistance of the zirconia frameworks (*p* < 0.001).

**Figure 3:**
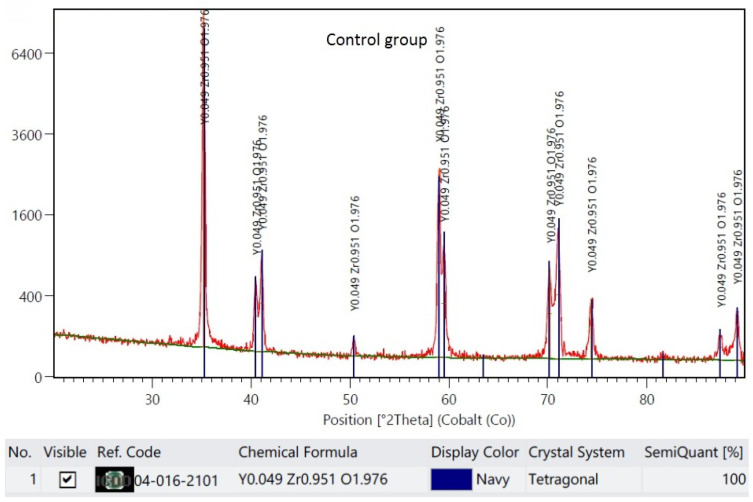
Representative plot of X-ray diffraction pattern for specimens with an untreated surface, the control group.

**Figure 4:**
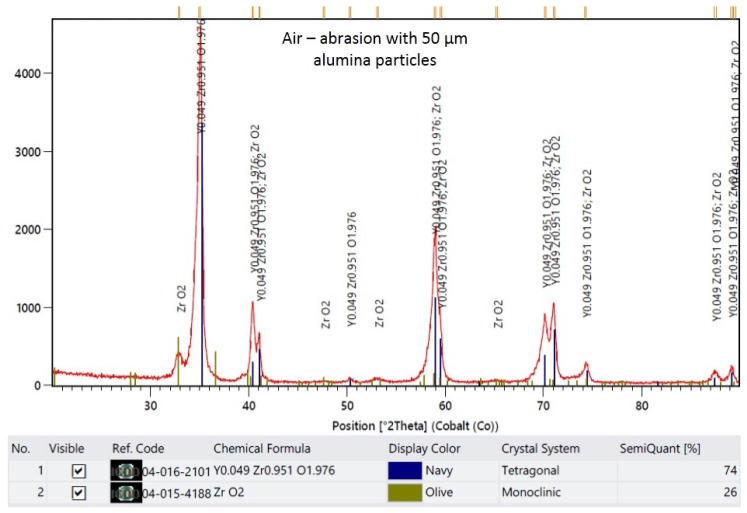
Representative plot of X-ray diffraction pattern for specimens that were air-abraded with 50 µm alumina particles.

**Figure 5:**
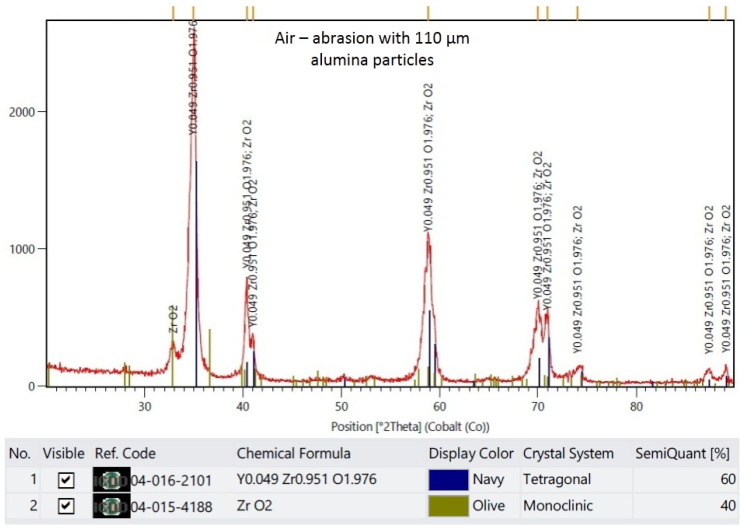
Representative plot of X-ray diffraction pattern for specimens that were air-abraded with 110 µm alumina particles.

**Figure 6:**
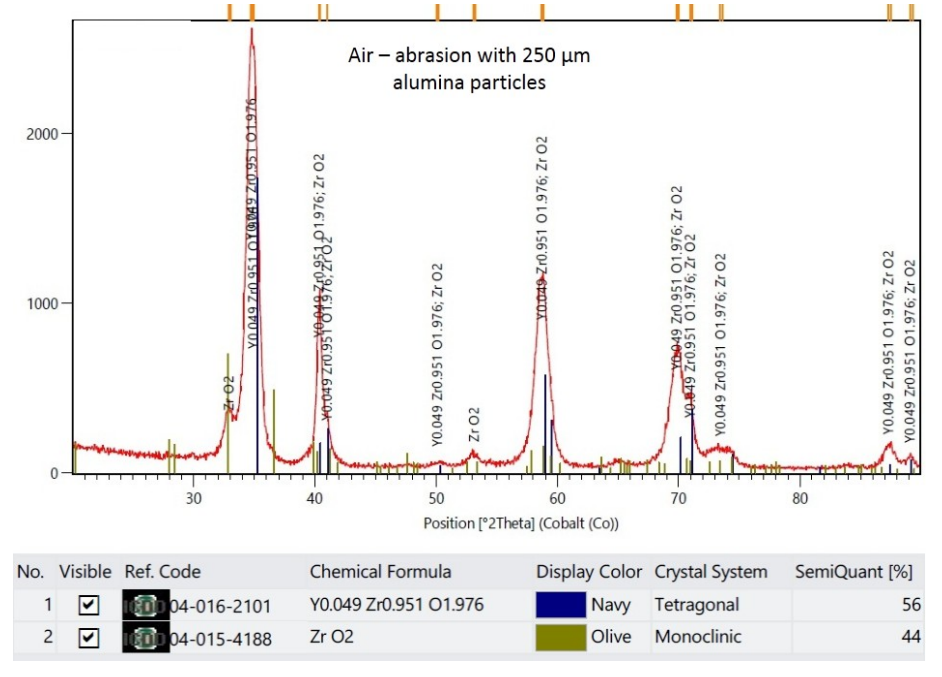
Representative plot of X-ray diffraction pattern for specimens that were air-abraded with 250 µm alumina particles.

The XRD results are demonstrated in representative plots in Figure 3, Figure 4, Figure 5 and Figure 6. X-ray diffractograms are the graphic record of measurements presented as a rectangular coordinate system, in which the X-axis refers to the magnitude of the diffraction angle and the Y-axis refers to relative intensity of peaks. In the control group, in which the specimen surface was untreated, the monoclinic phase was not detected. Meanwhile, in groups in which the air abrasion was used, the monoclinic phase was detected, and its content increased with the increase of the alumina particle size. The mean monoclinic volume fraction reached the following respective values: for air abrasion with 50 µm alumina particles, the *m* content was 26%; for 110 µm, it was 40%; and for 250 µm, it was 44%.

## 4. Discussion

This study rejected the null hypothesis that the airborne-particle abrasion with alumina particles of different sizes does not affect the fracture resistance of zirconia frameworks and the phase transformation induction in the surface layers of zirconia-based ceramics. The obtained results indicate that performing alumina air abrasion of the inner surface of zirconia frameworks results in a double increase of their fracture resistance. Moreover, the increase in the size of alumina particles entails an increase in the fracture resistance of zirconia frameworks. It is strongly related to the phase transformation toughening phenomenon. The XRD revealed that the air abrasion induced the tetragonal-to-monoclinic phase formation.

In this study, the fracture resistance of zirconia-based ceramics frameworks was assessed by subjecting the specimens to a load until they fractured. There are different shapes of zirconia specimens recommended for this test in the literature on f.e. cylindrical specimens, specimens in the shape of FPRs (fixed prosthetic restorations), such as single crowns or bridges, cemented on abutments made of metal or other materials, with or without the imitation of periodontal ligaments, where the load is applied along the long axis or at different angles [36,37,38,39,40,41]. In this study, the specimens were designed as frameworks (with walls of uniform thickness of 0.5 mm) without porcelain veneering, so that they would resemble the features of zirconia restorations used in vivo. In the literature, the mechanical strength of 3Y-TZP after airborne-particle abrasion was assessed also with the biaxial flexural strength test (BFS). The conclusions were similar to the results obtained in the current study. However, in the current study, the influence of air abrasion with different sizes of alumina particles under the same research conditions was examinedin one study, so that the results are comparable. Kosmač et al. demonstrated that the biaxial flexural strength of zirconia increases significantly after performing air abrasion with 110 µm alumina particles (at a pressure of 4 bar) in comparison with the untreated specimens (1239 MPa vs. 1021 MPa and 1224 MPa vs. 914 MPa) [42,43]. Cotič et al. indicate that the biaxial flexural strength of zirconia increases by 40% when air abrasion (110 µm Al_2_O_3_, at a pressure of 2.5 bar) is performed in comparison with the untreated specimens (1458.8 vs. 1015.9 MPa) [31]. Moqbel et al. indicate that biaxial flexural strength of zirconia increases after air abrasion with 50 µm alumina (0.1 MPa pressure, BFS 1153 MPa and 0.25 MPa, BFS 1137 MPa) in comparison with the untreated specimens (720 MPa) [44]. Also, Sato et al. proved that alumina air abrasion entails the increase in the biaxial flexural strength of zirconia. Moreover, they found a correlation between the monoclinic phase volume increase and the increase of the mechanical strength of zirconia [20]. Kelch et al. indicate that air abrasion of the zirconia surface significantly increases the flexural strength of zirconia. Also, the increase in alumina particle size (50 µm vs. 105 µm) increases the flexural strength of 3Y-TZP and the monoclinic phase content [45]. Wongkamhaeng et al. found that the flexural strength values of 3Y-TZP specimens that were air-abraded with 50 µm alumina were higher (1662.6 MPa) than for 250 µm alumina (1371.4 MPa) [46]. Chintapalli et al. demonstrated that the use of air abrasion with 110 µm alumina particles (at a 2-bar pressure) results in the increase of the biaxial flexural strength of zirconia. Meanwhile, air abrasion with 250 µm alumina particles decreased the mechanical strength of zirconia-based ceramics. Air abrasion was performed at a 90° angle in this case, while the air abrasion at a 30° angle had only a mild influence on the mechanical strength of zirconia, irrespective of the alumina particle size [47]. On the other hand, Karakoca et al. did not find any significant difference between alumina that were air-abraded zirconia specimens (110 µm Al_2_O_3_, at a pressure of 4 bar) and the untreated specimens [48]. The aforementioned researchers explain the increase of the mechanical strength of zirconia-based ceramics after alumina air abrasion with the increase of the monoclinic phase volume and the PTT phenomenon.

The results of the XRD test obtained in the current investigation demonstrate that alumina air abrasion of the zirconia-based ceramics’ surface induces the phase transformation and the increase of the monoclinic phase volume. Such results are similar to those obtained by the other research studies, in which the monoclinic phase was also detected after performing the alumina air abrasion. Karakoca et al. detected a 9.44–14.5% monoclinic phase volume in the surface layers of zirconia after air abrasion with 110 µm alumina particles [48]. Moqbel et al. indicate a 7.5–10.4 vol % monoclinic phase ratio after air abrasion with 50 µm alumina (at a pressure of 0.1 MPa and 0.25 MPa) [44]. Kosmač et al. found a 13.9–15.2% monoclinic phase volume increase [43]. Guazzato et al. found a 9.5% [16] and a 13.9% monoclinic phase volume increase [42]. Sato et al. found a 4.5% monoclinic phase volume increase after air abrading of the zirconia surface with 70 µm alumina particles [20], while Moon et al. found an 11.4% monoclinic phase volume increase [49]. In other research studies, a monoclinic phase volume increase after alumina air abrasion was also confirmed: 6% (50 µm alumina particles) [50], 8.68% (150 µm alumina particles) [51], and 4% [18].

In the present study, we compared three different sizes of alumina particles used for air abrasion and their influence on the phase content in the zirconia surface—it is clear that the alumina particle size used for air abrasion significantly affects the monoclinic phase volume detected in the topmost layer of 3Y-TZP. Such observations are confirmed by Hallmann et al., Monaco et al., and Kelch et al. [19,45,52,53]. Hallmann et al. compared the influence of air abrasion of 3Y-TZP with 50 µm and 110 µm alumina particles on the monoclinic phase volume, and it was 5.16% and 7.92%, respectively [19]. In other studies conducted by these researchers, they demonstrated a 3.99% monoclinic phase volume increase when zirconia specimens were air-abraded with 50 µm alumina particles and a 6.46% increase for 110 µm alumina particles [53]. Monaco et al. detected a 10% monoclinic phase volume increase for zirconia that were air-abraded with 50 µm alumina particles and a 14% increase for 110 µm alumina particles [52]. Chen et al. detected 7.3% of a monoclinic fraction and 14.5% depending on the 3Y-TZP manufacturer [54]. On the other hand, Chintapalli et al. found that the size of alumina particles used for the air abrasion of zirconia is insignificant for the monoclinic phase volume value [17]. They showed that air abrasion with 110 µm and 250 µm alumina particles of the zirconia surface resulted in a 12–15% monoclinic phase volume increase, irrespective of the particle size [17]. Kim et al. found that air abrasion produced a small monoclinic phase fraction (0–2.3 wt%) with the highest value of 2.3 wt% observed for specimens that were air-abraded with 125 µm alumina (they compared five sizes of alumina particles: 25 µm, 50 µm, 90 µm, 110 µm, and 125 µm at a 0.2 MPa pressure) [55].

The monoclinic phase volume present on the superficial layers of 3Y-TZP is a relatively small fraction; the exact transformation depth is bigger. In theresearch conducted, the examined maximum depth of zirconia was 21 µm, while in Chintapalli et al.’s studies, the penetration depth of the X-rays was 3 µm. This may be the reason for the higher values of the monoclinic phase volume obtained in the current study [17]. Chintapalli et al. proved that the total transformation depth is 12 ± 1 µm for a 110 µm alumina particle abrasion and 13 ± 1 µm for a 250 µm alumina particle air abrasion [17]. Cotič et al. showed that the transformation zone depth is about 10 µm. They observed that the transformation zone is not homogenous. The tetragonal-to-monoclinic ratio and the microstructure observed on the FIB-prepared cross-sections changes with different depths. They demonstrated that within the topmost 1 µm, severe plastic deformation is seen with only a subtle outline of the zirconia grains. At a depth between 1 and 6 µm of the zirconia specimen, deformed zirconia grains were observed with more distinct boundaries, and new domains were seen. At 10 µm, they observed less severe alterations [31]. On the other hand, Wongkamhaeng et al. assessed the depth of the phase transformation as 14.5 ± 1.2 µm (air abrasion with 50 µm alumina) and 47.2 ± 3.0 µm (250 µm alumina) [46]. Kim et al. indicate that the deepest transformation layer did not exceed the 2.9 µm distance (they compared five grades of alumina particles: 25 µm, 50 µm, 90 µm, 110 µm, and 125 µm at a 0.2 MPa pressure) [55].

In the current study, it is demonstrated that the air abrasion with alumina particles of the surface of zirconia results in the increase of the mechanical strength of this material and the increase in the monoclinic phase content. Such observations are confirmed by the other authors. The rise of the mechanical strength of 3Y-TZP is explained as the result of compressive stresses that occur within the topmost layer of zirconia specimens as a result of the 4–5% monoclinic crystal volume increase [20,43,48]. As the airborne alumina particle abrasion used to be considered as the detrimental treatment for 3Y-TZP, although it has also many advantages, recent studies reject those opinions. Alumina air abrasion entails the superficial erosion of 3Y-TZP and induces the tetragonal-to-monoclinic phase transformation, leading to a PTT or an LTD phenomenon [52]. The microstructure alterations may affect the mechanical parameters and reliability of zirconia-based restorations. However, Cotič et al. demonstrated that the LTD annihilates the stresses present at the topmost layer of 3Y-TZP and gradually leads to a decrease of the PTT zone. As a result, the strength of zirconia decreases and finally reaches the values measured prior to alumina particle air abrasion [31]. They found that the speed of LTD equates with the speed of the LTD of as-sintered zirconia specimens (untreated). Also, a prolonged time of in vitro LTD does not affect the strength of air-abraded specimens in comparison with the as-sintered ones [31]. Cotič et al. conclude that alumina particle air abrasion does not affect the zirconia restorations’ reliability and stability during the clinical service. If the appropriate air abrasion parameters are applied, then such treatment should not be harmful to the material’s properties over time [31]. Moreover, Inokoshi et al. revealed that air abrasion with alumina particles of the zirconia surface increases the zirconia resistance to the LTD phenomenon when compared to the as-sintered zirconia specimens [32]. Kelch et al. also observed that aging of zirconia specimens resulted in higher flexural strength values for air-abraded specimens [45].

Assuming all the results obtained in this study, the alumina airborne-particle abrasion of 3Y-TZP results in an increase of the monoclinic phase volume in the topmost layers of zirconia and in an increase of the fracture resistance of zirconia-based frameworks. An increase of the size of alumina particles entailed an increase of the monoclinic phase volume and an increase of the mechanical strength of 3Y-TZP. According to the aforementioned research studies conducted by Cotič et al. and Inokoshi et al., alumina airborne-particle abrasion does not affect the reliability and stability of 3Y-TZP restorations over time despite the rise in the monoclinic phase content present in the alumina air-abraded specimens [31,32]. Nevertheless, it is still unknown what volume of the monoclinic phase can be still considered as harmless. Also, it must be noted that the tetragonal-to-monoclinic phase transformation is irreversible in the mouth environment. Therefore, we conclude that the most favorable proceeding would be performing the airborne-particle abrasion with alumina particles of such size which causes a phase transformation in the minimal extent and still ensures a high bond strength of 3Y-TZP to resin cements. We conclude that the use of 50 µm alumina particles for the air abrasion of zirconia-based dental ceramics would be the most favorable option to enhance the bond strength of zirconia to resin cements, because such a proceeding has a mild influence on the t–m transformation in comparison with bigger alumina particles and still increases the fracture resistance of zirconia-based frameworks.

## 5. Conclusions

1. Alumina airborne-particle abrasion of the zirconia surface results in an increase in the fracture resistance of zirconia-based dental ceramics frameworks. The fracture resistance increases with an increase of the alumina particle size.
2. Alumina airborne-particle abrasion of the zirconia surface induces a phase transformation in the surface layers of zirconia. An increase of the alumina particle size entails an increase of the monoclinic phase presence in the surface layers of zirconia.

## Data Availability

Not applicable.

## References

[1] Conrad H.J., Seong W.-J., Pesun I.J. (2007). Current ceramic materials and systems with clinical recommendations: A systematic review. J. Prosthet. Dent..

[2] Borges G.A., Sophr A.M., de Goes M.F., Sobrinho L.C., Chan D.C.N. (2003). Effect of etching and airborne particle abrasion on the microstructure of different dental ceramics. J. Prosthet. Dent..

[3] Thompson J.Y., Stoner B.R., Piascik J.R., Smith R. (2011). Adhesion/cementation to Zirconia and other non-silicate ceramics: Where are we now?. Dent. Mater..

[4] Blatz M.B., Phark J.-H., Ozer F., Mante F.K., Saleh N., Bergler M., Sadan A. (2010). In vitro comparative bond strength of contemporary self-adhesive resin cements to zirconium oxide ceramic with and without air-particle abrasion. Clin. Oral. Investig..

[5] Akin H., Ozkurt Z., Kirmali O., Kazazoglu E., Ozdemir A.K. (2011). Shear bond strength of resin cement to Zirconia ceramic after aluminum oxide sandblasting and various laser treatments. Photomed. Laser Surg..

[6] Piascik J.R., Swift E.J., Thompson J.Y., Grego S., Stoner B.R. (2009). Surface modification for enhanced silanation of zirconia ceramics. Dent. Mater..

[7] Aboushelib M.N., Kleverlaan C.J., Feilzer A.J. (2007). Selective infiltration-etching technique for a strong and durable bond of resin cements to zirconia-based materials. J. Prosthet. Dent..

[8] Blatz M.B., Sadan A., Martin J., Lang B. (2004). In vitro evaluation of shear bond strengths of resin to densely sintered high-purity zirconium-oxide ceramic after long-term storage and thermal cycling. J. Prosthet. Dent..

[9] Yoshida K., Tsuo Y., Atsuta M. (2006). Bonding of dual-cured resin cement to zirconia ceramic using phosphate acid estermonomer and zirconate coupler. J. Biomed. Mater. Res. B Appl. Biomater..

[10] Łagodzińska P., Bociong K., Sokołowski J., Dejak B. (2019). Comparative study of the shear bond strength of zirconia-based dental ceramics to resin cements after chemo-mechanical surface modification. J. Stomatol..

[11] Gomes A.L., Castillo-Oyague R., Lynch C.D., Montero J., Albaladejo A. (2013). Influence of sandblasting granulometry and resin cement composition on microtensile bond strength to zirconia ceramic for dental prosthetic Framework. J. Dent..

[12] Phark J.-H., Duarte S., Blatz M., Sadan A. (2009). An in vitro evaluation of the long-term resin bond to a new densely sintered high-purity zirconium-oxide ceramic surface. J. Prosthet. Dent..

[13] Yun J.-y., Ha S.-r., Lee J.-b., Kim S.-h. (2010). Effect of sandblasting and various metal primers on the shear bond strength of resin cement to Y-TZP ceramic. Dent. Mater..

[14] Yang B., Barloi A., Kern M. (2010). Influence of air-abrasion on zirconia ceramic bonding using an adhesive composite resin. Dent. Mater..

[15] Quaas A.C., Yang B., Kern M. (2007). Panavia F 2.0 bonding to contaminated zirconia ceramic after different cleaning procedures. Dent. Mater..

[16] Guazzato M., Quach L., Albakry M., Swain M.V. (2005). Influence of surface and heat treatments on the flexural strength of Y-TZP dental ceramic. J. Dent..

[17] Chintapalli R.K., Marro F.G., Jimenez-Pique E., Anglada M. (2013). Phase transformation and subsurface damage in 3Y-TZP after sandblasting. Dent. Mater..

[18] Zhang Y., Lawn B.R., Rekow E.D., Thompson V.P. (2004). Effect of sandblasting on the long term performance of dental ceramics. J. Biomed. Mater. Res. B Appl. Biomater..

[19] Hallmann L., Ulmer P., Reusser E., Hammerle C.H.F. (2012). Surface characterization of dental Y-TZP ceramic after air abrasion treatment. J. Dent..

[20] Sato H., Yamadad K., Pezzotti G., Nawa M., Ban S. (2008). Mechanical properties of dental zirconia ceramics changed with sandblasting and heat treatment. Dent. Mater. J..

[21] Śmielak B., Klimek L. (2018). Effect of air abrasion on the number of particles embedded in Zirconia. Materials.

[22] Denry I., Kelly J.R. (2008). State of the art of zirconia for dental applications. Dent. Mater..

[23] Heuer A.H., Lange F.F., Swain M.V., Evans A.G. (1986). Transformation toughening: An overview. J. Am. Ceram. Soc..

[24] Garvie R.C., Nicholson P.S. (1972). Phase analysis in zirconia systems. J. Am. Ceram. Soc..

[25] Garvie R.C., Hannink R.H., Pascoe R.T. (1975). Ceramic steel?. Nature.

[26] Kelly J.R., Denry I. (2008). Stabilized zirconia as a structural ceramic: An overview. Dent. Mater..

[27] Lughi V., Sergo V. (2010). Low temperature degradation—Aging of zirconia: A critical review of the relevant aspects in dentistry. Dent. Mater..

[28] Swab J.J. (1991). Low temperature degradation of Y-TZP materials. J. Mater. Sci..

[29] Basu B., Vleugels J., Van Der Biest O. (2004). Microstructure-toughness-wear relationship of tetragonal zirconia ceramics. J. Eur. Ceram..

[30] Liu D., Matinlinna J.P., Tsoi J.K.-H., Pow E.H.N., Miyazaki T., Shibata Y., Kan C.-W. (2013). A new modified laser pretreatment for porcelain zirconia bonding. Dent. Mater..

[31] Cotic J., Jevnikar P., Kocjan A. (2017). Ageing kinetics and strength of airborne-particle abraded 3Y-TZP ceramics. Dent. Mater..

[32] Inokoshi M., Vanmeensel K., Zhang F., De Munck J., Eliades G., Minakuchi S., Naert I., Meerbeek B., Vleugels J. (2015). Aging resistance of surface–treated dental zirconia. Dent. Mater..

[33] Kern M. (2010). Controlled airborne-particle abrasion of zirconia ceramic restorations. J. Prosthet. Dent..

[34] Attia A., Lehmann F., Kern M. (2011). Influence of surface conditioning and cleaning methods on resin bonding to zirconia ceramic. Dent. Mater..

[35] Karimi M., Asefnejad A., Aflaki D., Surendar A., Baharifar H., Saber-Samandari S., Khandan A., Khan A., Toghraie D. (2021). Fabrication of shapeless scaffolds reinforced with baghdadite-magnetite nanoparticles using a 3D printer and freeze-drying technique. J. Mater. Res. Technol..

[36] Tinschert J., Natt G., Mautsch W., Augthun M., Spiekermann H. (2001). Fracture resistance of Lithium Dissilicate-, Alumina- and Zirconia-Based three-unit fixed partial dentures: A laboratory study. Int. J. Prosthodont..

[37] Sundh A., Molin M., Sjogren G. (2005). Fracture resistance of yttrium oxide partially-stabilized zirconia all-ceramic bridges after veneering and mechanical fatigue testing. Dent. Mater..

[38] Kim J.H., Park J.H., Park Y.B., Moon H.S. (2012). Fracture load of zirconia crowns according to the thickness and marginal design of coping. J. Prosthet. Dent..

[39] Tsalouchou E., Cattell M.J., Knowles J.C., Pittayachawan P., McDonald A. (2008). Fatigue and fracture properties of yttria partially stabilized zirconia crown systems. Dent. Mater..

[40] Att W., Grigoriadou M., Strub J.R. (2007). ZrO_2_ three-unit fixed partial dentures: Comparison of failure load before and after exposure to a mastication symulator. J. Oral. Rehabil..

[41] Luthy H., Filser F., Loeffel O., Schumacher M., Gauckler L.J., Hammerle C.H.F. (2005). Strehgth and relability of four-unit all-ceramic posterior bridges. Dent. Mater..

[42] Kosmac T., Oblak C., Jevnikar P., Funduk N., Marion L. (1999). The effect of Surface grinding and sandblasting on flexural strength and reliability of 3Y-TZP zirconia ceramic. Dent. Mater..

[43] Kosmac T., Oblak C., Jevnikar P., Funduk N., Marion L. (2000). Strength and reliability of Surface treated Y-TZP dental ceramics. J. Biomed. Mater Res. B Appl. Biomater..

[44] Moqbel N.M., Al-Akhali M., Wille S., Kern M. (2020). Influence of Aging on Biaxial Flexural Strength and Hardness of Translucent 3Y-TZP. Materials.

[45] Kelch M., Schulz J., Edelhoff D., Sener B., Stawarczyk B. (2019). Impact of different pretreatments and aging procedures on the flexural strength and phase structure of zirconia ceramics. Dent. Mater..

[46] Wongkamhaeng K., Dawson D.V., Holloway J.A., Denry I. (2019). Effect of Surface Modification on In-Depth Transformations and Flexural Strength of Zirconia Ceramics. J. Prosthodont..

[47] Chintapalli R.K., Rodriguez A.M., Marro F.G., Anglada M. (2014). Effect of sandblasting and residual stress on strength of zirconia for restorative dentistry applications. J. Mech. Behav. Biomed. Mater..

[48] Karakoca S., Yilmaz H. (2009). Influence of surface treatments on surface roughness, phase transformation, and biaxial flexural strength of 3Y-TZP ceramics. J. Biomed. Mater. Res. B Appl. Biomater..

[49] Moon J., Kim S., Lee J., Ha S., Choi Y. (2011). The effect of preparation order on the crystal structure of yttria-stabilized tetragonal zirconia polycrystal and the shear bond strength of dental resin cements. Dent. Mater..

[50] Guess P.C., Zhang Y., Kim J.W., Rekow E.D., Thompson V.P. (2010). Damage and reliability of Y-TZP after cementation surface treatment. J. Dent. Res..

[51] Hallmann L., Ulmer P., Wille S., Polomskyi O., Kobel S., Trottenberg T., Bornholdt S., Haase F., Kersten H., Kern M. (2016). Effect of surface treatments on the properties and morphological change of dental zirconia. J. Prosthet. Dent..

[52] Monaco C., Tucci A., Esposito L., Scotti R. (2013). Microstructural changes produces by abrading y-TZP in presintered and sintered conditions. J. Dent..

[53] Hallmann L., Ulmer P., Reusser E., Hammerle C.H.F. (2012). Effect of blasting pressure, abrasive particle size and grade on phase transformation and morphological change of dental zirconia surface. Surf. Coat. Technol..

[54] Chen B., Yan Y., Xie H., Meng H., Zhang H., Chen C. (2020). Effects of Tribochemical Silica Coating and Alumina-Particle Air Abrasion on 3Y-TZP and 5Y-TZP: Evaluation of Surface Hardness, Roughness, Bonding, and Phase Transformation. J. Adhes. Dent..

[55] Kim H.-K., Yoo K.-W., Kim S.-J., Jung C.-H. (2021). Phase Transformations and Subsurface Changes in Three Dental Zirconia Grades after Sandblasting with Various Al_2_O_3_ Particle Sizes. Materials.

